# Antitumor Activity of Prosopis glandulosa Torr. on Ehrlich Ascites Carcinoma (EAC) Tumor Bearing Mice

**Published:** 2011

**Authors:** Raju Senthil Kumar, Balasubramanian Rajkapoor, Perumal Perumal, Thangavel Dhanasekaran, Manonmani Alvin Jose, Chennakesavalu Jothimanivannan

**Affiliations:** a *Department of Pharmaceutical Chemistry, Swamy Vivekanandha College of Pharmacy, Tiruchengodu-637 205, Namakkal (Dt), Tamilnadu, India.*; b *Department of Pharmacology, Dayanandha Sagar College of Pharmacy, Bangalore-560 078, India.*; c *Department of Pharmaceutical Chemistry, JKK Nataraja College of Pharmacy, Komarapalayam, Namakkal Dt, Tamilnadu, India.*

**Keywords:** *Prosopis glandulosa*, Ehrlich Ascites Carcinoma, Life Span, Hematological Parameters, Solid tumor

## Abstract

The antitumor activity of ethanol extract of *Prosopis glandulosa *Torr. (EPG) was evaluated against Ehrlich ascites carcinoma (EAC) tumor model in Swiss albino mice on dose dependent manner. The activity was assessed using survival time, average increase in body weight, hematological parameters and solid tumor volume. Oral administration of EPG at the dose of 100, 200 and 400 mg/Kg, significantly (p < 0.001) increased the survival time and decreased the average body weight of the tumor bearing mice. After 14 days of inoculation, EPG was able to reverse the changes in the hematological parameters, protein and PCV consequent to tumor inoculation. Oral administration of EPG was effective in reducing solid tumor mass development induced by EAC cells. The results indicate that EPG possess significant antitumor activity on dose dependent manner.

## Introduction

Cancer is one of the leading causes of mortality worldwide and the failure of conventional chemotherapy to affect major reduction in the mortality indicates that new approaches are critically needed. The new and recent approaches of chemotherapy serve as an attractive alternative to control the cancer (1). Recently, the major focus of research in chemotherapy for cancer includes the identification, characterization and development of new and safe cancer chemopreventive agents. A large number of agents including natural and synthetic compounds have been identified as having some potential cancer chemotherapeutic value (2). A number of natural products have been studied for anticancer activity on various experimental models. This has resulted in the availability of nearly 30 effective anticancer drugs (3).

Natural products are playing an important role as a source of effective anticancer agents and it is significant that 60% of currently used anticancer agents are derived from natural sources, including plants, marine organism and micro-organism (4, 5). The mechanism of interaction between many secondary metabolites and cancer cells has been studied extensively (6). In particular, there is growing interest in the pharmacological evaluation of various plants used in Indian traditional system of medicine. Plant-derived natural products like flavonoids, steroids, alkaloids and terpenoids have received considerable attention in recent years due to their diverse pharmacological activities, including antioxidant and anticancer activity (7, 8). Antioxidants play an important role in inhibiting and scavenging radicals and thus, protecting humans against infection and degenerative diseases.

*Prosopis glandulosa *Torr. (Mimosaceae) is a small to medium height tree or shrub that is thorny and branching near the ground found mostly in southern parts of India. It is popularly known as Seemai Parambai or Vanni in Tamil. The bark and leaves are used by the tribes and native medical practitioners to treat various ailments such as leprosy, dysentery, bronchitis, asthma, leucoderma, piles, tremors of the muscles, tumors, eye diseases and rheumatism (9, 10). Literature studies have indicated that the plant contains flavan-3-ol dimmer, mesquitol-(5-8)-catechin (11). Oleanolic acid isolated from the leaves and twigs of *Prosopis glandulosa *was identified as an anti-HIV principle (12). The plant also contains alkaloids (13). A new potent antiinfective and antiparasitic 2, 3-dihydro-1H-indolizinium chloride was isolated from *Prosopis glandulosa *(14). The present study was carried out to evaluate the antitumor activity of ethanol extract of leaves of *Prosopis glandulosa *(EPG) against Ehrlich ascites carcinoma (EAC) in mice.

## Experimental

*Collection and extraction*

The fresh leaves of *Prosopis glandulosa *were collected in and around Tiruchengodu in Namakkal district, Tamilnadu, India, in June 2007 and authenticated by Dr. G. V. Murthy, Botanical Survey of India, Coimbatore, Tamilnadu, India. A voucher specimen (Voucher No. PCH 004) representing this collection has been retained in our laboratory for future reference.

The leaves were shade, dried and pulverized. The powder was treated with petroleum ether for dewaxing and removing chlorophyll. Later, it was packed (250 g) in soxhlet apparatus and subjected to continuous hot percolation for 8 h using 450 mL ethanol (75% v/v) as solvent. The ethanol extract was concentrated under vacuum and dried in a dessicator (yield 9.5 g, 3.7% w/w). Without any purification, aliquot portions of the crude extract were suspended in 5% gum acacia for use on each day of our experiment.

The Phytochemical studies were performed as described by Wagner et al. (15). The presence of alkaloids, glycosides, flavonoids, phenolic compounds, steroids and terpenoids were analyzed. The extract showed the positive test for alkaloids, glycosides, triterpenes, flavonoids and phenolic compounds.

*Animals*

Swiss male albino mice (20-25 g) were procured from Venkateshwara Enterprises (Bangalore, Karnataka, India) and used throughout the study. They were housed in microlon boxes in a controlled environment (with temperature of 25 ± 2°C and 12 h dark/light cycle) with standard laboratory diet and water *ad libitum*. The study was conducted after obtaining Institutional Animal Ethical Committee clearance (P.Ch. 002/2007-08).

*Acute toxicity studies (LD*_50_ *)*

The oral acute toxicity study of the extract was carried out in Swiss albino mice using up and down procedure as per OECD, 2001 (16). Mice received ethanol extract at various doses (500-2,000 mg/Kg) orally by gavage. They were observed for toxic symptoms continuously for the first 4 h after dosing. Finally, the number of survivors was noticed after 24 h. In the toxicity study, no mortality occurred within 24 h under the tested doses of EPG. 

*Cells*

EAC cells were originally obtained through the courtesy of Amala Cancer Research Center (Thrissur, Kerala, India). They were maintained by weekly intraperitoneal inoculation of 10^6^ cells/mouse (17).

*Effect of EPG on survival time*

Animals were inoculated with 1 × 10^6^ cells/mouse on day ‘0’ and the treatment with EPG started 24 h after inoculation, at doses of 100, 200 and 400 mg/Kg/day, *p.o. *The control group was treated with the same volume of 5% gum acacia solution. All the treatments were given for nine days. The median survival time (MST) and average body weight changes of each group, consisting of 6 mice, were noted. The antitumor efficacy of EPG was compared with that of 5-fluorouracil (Dabur Pharmaceuticals, India; 5-FU, 20 mg/Kg/day, IP for 9 days). The MST of the treated groups was compared with that of the control group using the following calculation:

Increase in life span = (T – C) / C × 100

T = number of days the treated animals survived 

C = number of days the control animals survived (18).

*Effect of EPG on hematological parameters*

In order to detect the influence of EPG on hematological status of EAC bearing mice, a comparison was made among five groups (n = 5) of mice on the 14^th ^day after inoculation. The groups were comprised of (I) Tumor bearing mice, (II) Tumor bearing mice treated with EPG (100 mg/Kg/day, p.o. for 9 days), (III) Tumor bearing mice treated with EPG (200 mg/Kg/day, p.o. for 9 days), (IV) Tumor bearing mice treated with EPG (400 mg/Kg/day, p.o. for 9 days), and (V) Control mice (normal). Blood was drawn from each mouse by the retroorbital plexus method and the white blood cells (WBC), red blood cells (RBC), hemoglobin, protein and packed cell volume (PCV) were determined (19-21).

*Effect of EPG on solid tumor*

Mice were divided into four groups (n = 6). Tumor cells (1 × 10^6^ cells/mouse) were injected into the right hind limb of all the animals intramuscularly. The mice of group I were served as control. Group II received EPG (100 mg/Kg/day, p.o.); group III received EPG (200 mg/Kg/day, p.o.) and group IV received EPG (400 mg/Kg/day, p.o.) for 5 alternative days. Tumor mass was measured from the 11^th^ day of tumor induction. The measurement was carried out every 5 days for a period of 30 days. The volume of tumor mass was calculated using the formula V = 4/3 πr^2^, where ‘r’ is the mean of ‘r^1^’ and ‘r^2^’ which are the two independent radii of the tumor mass (22).

*Statistical analysis*

All values were expressed as mean ± SEM. Statistical analysis was performed with one way analysis of variance (ANOVA) followed by Dunnett’s t-test. p-values < 0.05 were considered to be statistically significant when compared to control.

## Results and Discussion

*Effect of EPG on survival time*

The effect of EPG on the survival of tumor bearing mice is shown in Table 1. 

**Table 1:** Effect of EPG on median survival time and average increase in body weight of EAC tumor bearing mice

**Design of treatment**	**MST (in days)**	**Increase in life span (%)**	**Average increase in body weight (g)**
Tumor control	16 ± 0.75	-	13.3 ± 0.61
5-FU (20 mg/Kg, IP)	31 ± 0.41^*^	93.75	4.0 ± 0.44^*^
EPG (100 mg/Kg)	26 ± 0.92^**^	62.5	8.3 ± 0.84 ^*^
EPG (200 mg/Kg)	28 ± 0.76^*^	75	5.3 ± 0.66^*^
EPG (400 mg/Kg)	32 ± 0.21^*^	100	4.3 ± 0.36^*^

N = 6 animals in each group.Values are expressed as mean ± SEM.

^*^p < 0.001; ^**^p < 0.01 when compared with control. Data were analyzed by one-way ANOVA followed by Dunnett’s test

The MST of the control group was 16 ± 0.75 days, whereas it was 26 ± 0.92, 28 ± 0.76, 32 ± 0.21 and 31 ± 0.41 days for the groups treated with EPG (100, 200 and 400 mg/Kg) and 5-FU (20 mg/Kg) respectively. The increase in the life span of tumor bearing mice treated with EPG (100, 200 and 400 mg/Kg) and 5-FU was found to be 62.5%, 75%, 100% and 93.75% respectively.

The effect of EPG on the inhibition of average increase in body weight is shown in Table 1. The average weight gain of tumor bearing mice was 13.3 ± 0.61 g, whereas it was 8.3 ± 0.84, 5.3 ± 0.66, 4.3 ± 0.36 and 4.0 ± 0.44 g for the groups treated with EPG (100, 200 and 400 mg/Kg) and 5-FU (20 mg/Kg) respectively.

*Effect of EPG on hematological parameters*

Hematological parameters of tumor bearing mice on 14^th^ day showed significant changes compared to the normal mice (Table 2). The total WBC count, protein and PCV were found to increase with a reduction in the hemoglobin content of RBC. The differential count of WBC showed that the percentage of neutrophils increased while that of lymphocytes decreased. At the same time interval, EPG (100, 200 and 400 mg/Kg) treatment could change these parameters near to normal. Maximum alteration occurred in the EGC treatment at the dose of 400 mg/Kg.

**Table 2:** Effect of EPG on hematological parameters of EAC-bearing mice

**Design of treatment**		**Normal**	**Tumor control**	**EPG** **(100 mg/Kg)**	**EPG** **(200 mg/Kg)**	**EPG** **(400 mg/Kg)**
Hb (gm %)		16.33 ± 1.1	5.9 ± 0.26	10.3 ± 1.64	11.5 ± 0.72	14.4 ± 0.21^*^
*RBC *(10^6^ cells/mm^3^)		4.4 ± 0.2	2.7 ± 0.76	0.97 ± 3.7	4.05 ± 1.16	3.86 ± 0.21^*^
WBC (10^3^ cells/mm^3^)		6.5 ± 0.1	13.7 ± 1.72	9.4 ± 1.3	8.1 ± 1.1	9.42 ± 0.1^*^
Protein (mg %)		8.5 ± 0.22	12.4 ± 1.7	9.8 ± 1.1^***^	8.12 ± 0.9	8.33 ± 0.3^**^
PCV (mm)		16.6 ± 0.21	34.33 ± 2.45	29 ± 2.7^*^	24 ± 2.16^**^	17.3 ± 0.22^**^
Differential Count (%)	Lymphocytes	70.7 ± 1.1	60 ± 3.92	76 ± 3.64^*^	83 ± 4.74^*^	65.5 ± 0.21^*^
	Neutrophils	30.3 ± 0.21	38 ± 3.2	23 ± 1.36^**^	15 ± 1.82	27.8 ± 0.25^*^
	Monocytes	1 ± 0	1 ± 0	1 ± 0	2 ± 0	1 ± 0

N = 5 animals in each group. Values are expressed as mean ± SEM.

^ *^ p < 0.001; ^**^p < 0.01; ^***^p < 0.05 when compared with control. Data were analyzed by one-way ANOVA followed by Dunnett’s test.

*Effect of EPG on solid tumor*

There was significant reduction in the tumor volume of mice treated with EPG (100, 200 and 400 mg/Kg). Tumor volume of control animals was 6.62 ± 0.38 mL whereas it was 4.26 ± 0.18, 4.21 ± 0.1 and 4.17 ± 0.21 mL for the groups treated with EPG (100, 200 and 400 mg/Kg) respectively (Table 3).

**Table 3:** Effect of EPG on solid tumor volume

**Design of treatment**	**Solid tumor volume ( mL)**				
	**15** ^th ^ **day**	**20** ^th^ **day**		**25** ^th^ **day**	**30** ^th ^ **day**
Tumor control	3.99 ± 0.23	4.63 ± 0.26	5.13 ± 0.41		6.62 ± 0.38
EPG (100 mg/Kg, p.o)	2.56 ± 0.28^*^	3.09 ± 0.51^*^	3.13 ± 0.68^*^		4.26 ± 0.18^*^
EPG (200 mg/Kg, p.o)	2.13 ± 0.21^*^	3.58 ± 0.16^*^	3.86 ± 0.21^*^		4.21 ± 0.1^*^
EPG (400 mg/Kg, p.o)	2.27 ± 0.37^*^	3.27 ± 0.37^*^	3.66 ± 0.19^*^		4.17 ± 0.21^*^

N = 6 animals in each group. Values are expressed as mean ± SEM.

^*^p < 0.001when compared with control. Data were analyzed by one-way ANOVA followed by Dunnett’s test.

Murine type tumors are common malignant tumors. The primary therapy for those tumors includes surgery, radiation therapy and chemotherapy. Although these therapies have been extremely successful in the treatment of early carcinoma, the prognosis for advanced and recurrent diseases remains extremely guarded. Some treatments produce serious side effects. 

The reliable criteria for judging the value of any anticancer drug are the prolongation of life span inhibition of gain in average body weight and the decrease in WBC (23, 24). The results of the present study showed an antitumor effect of EPG against EAC in Swiss albino mice. A significant (p < 0.001 and 0.05) enhancement of MST and decrement of gain in average body weight was observed.

There was a regular and rapid increase in ascetic fluid volume of EAC bearing mice. Ascitic fluid is the direct nutritional source of tumor growth; it meets the nutritional requirements of tumor cells (25). EPG treatment decreased the volume of solid tumor and increased the lifespan. 

The most common problems encountered in cancer chemotherapy are myelosuppression and anemia (26, 27). Anemia occurred in tumor bearing mice is mainly due to the reduction of RBC or hemoglobin production and this may occur either due to the iron deficiency or to hemolytic or other myelopathic conditions (28). The analysis of the hematological parameters showed minimum toxic effect in the mice treated with EPG. After 14 days of transplantation, EPG was able to reverse the changes in the hematological parameters consequent to tumor inoculation. This indicates that EPG has a protective effect on the hemopoietic system. 

The tumor volume of EPG treated mice shows significant reduction, which was observed on 30^th^ day. The maximum inhibition was produced by EPG at the dose of 400 mg/Kg. The reduction in solid tumor volume indicated that EPG plays a direct role in killing the tumor cells and enhances the curative effect of tumor chemotherapy. 

The antitumor activity of EPG was comparable to that of 5-fluorouracil which is commonly used as an active antitumor agent in vast series of clinical and preclinical studies (29). 

Preliminary phytochemical screening of the extract showed the presence of triterpenes, alkaloids and flavonoids. Flavonoids have been found to possess antimutagenic and antimalignant effects (30, 31). Moreover, they have a chemopreventive role in cancer through their effects on signal transduction in cell proliferation (32) and angiogenesis (33). The antitumor properties of the extracts may be due to these compounds. The present study points to the potential anticancer activity of *Prosopis glandulosa *that might be a promising chemotherapeutic agent against murine tumors. Further studies are in progress to characterize the active principles and to elucidate the mechanism of action.
